# Inhibition of Transforming Growth Factor-Activated Kinase 1 (TAK1) Blocks and Reverses Epithelial to Mesenchymal Transition of Mesothelial Cells

**DOI:** 10.1371/journal.pone.0031492

**Published:** 2012-02-27

**Authors:** Raffaele Strippoli, Ignacio Benedicto, Maria Luisa Perez Lozano, Teijo Pellinen, Pilar Sandoval, Manuel Lopez-Cabrera, Miguel Angel del Pozo

**Affiliations:** 1 Integrin Signaling Laboratory, Department of Vascular Biology and Inflammation, Centro Nacional de Investigaciones Cardiovasculares (CNIC), Madrid, Spain; 2 Bambino Gesù Children's Hospital, Rome, Italy; 3 Unidad de Biología Molecular, Instituto de Investigación Sanitaria Princesa (IP), Hospital Universitario de la Princesa, Madrid, Spain; 4 CIBER-ehd, Instituto de Salud Carlos III, Madrid, Spain; 5 Centro de Biología Molecular Severo Ochoa, CSIC-UAM, Cantoblanco, Madrid, Spain; University of Pittsburgh, United States of America

## Abstract

Peritoneal fibrosis is a frequent complication of peritoneal dialysis following repeated low grade inflammatory and pro-fibrotic insults. This pathological process may lead to ultrafiltration failure and eventually to the discontinuing of the therapy. Fibrosis is linked to epithelial to mesenchymal transition (EMT) of the peritoneal mesothelial cells, which acquire invasive and fibrogenic abilities. Here, we analyzed the role of the transforming growth factor-activated kinase-1 (TAK1) in the EMT of primary mesothelial cells from human peritoneum. The inhibition of TAK1 in mesenchymal-like mesothelial cells from the effluents of patients undergoing peritoneal dialysis led to the reacquisition of the apical to basolateral polarity, to increased expression of epithelial and to down-regulation of mesenchymal markers. TAK1 inhibition also resulted in decreased migratory/invasive abilities of effluent-derived mesothelial cells. Simultaneous inhibition of ERK1/2 and TAK1 pathways did not lead to an additive effect in the reacquisition of the epithelial phenotype. Inhibition of TAK1 also blocked EMT *in vitro* and reduced the levels of PAI-1, which is involved in fibrosis and invasion. Analysis of signalling pathways downstream of TAK1 involved in EMT induction, showed that TAK1 inhibition reduced the transcriptional activity of NF-κB and Smad3, as well as the phosphorylation of c-jun, while enhancing Smad1–5–8 activity. These results demonstrate that TAK1 is a cross-point in a network including different pro-EMT transcription factors, such as NF-κB, Snail, AP-1 and Smads. The identification of TAK1 as a main biochemical mediator of EMT and fibrosis in mesothelial cells from human peritoneum and the study of signalling pathways induced by its activity may be relevant in the design of new therapies aimed to counteract peritoneal fibrosis.

## Introduction

The main complication of the peritoneal dialysis is the instauration of peritoneal fibrosis. This process is associated with the progressive decrease of the dialytic function of the peritoneal membrane and eventually with the discontinuing of the therapy [1]. The establishment of peritoneal fibrosis has been associated with the epithelial to mesenchymal transition (EMT) of the peritoneal mesothelial cells monolayer [2]. Repeated low grade inflammatory stimuli may induce peritoneal mesothelial cells to lose intercellular junctions, to undergo morphological changes and to progressively acquire invasive, fibrogenic and angiogenic abilities [1]. EMT and fibrosis of the peritoneal membrane have similarities with analogous inflammation-related pathologic alterations of other tissues and organs, occurring in liver, heart, the pleural membrane, and in the kidney [3], [4]. EMT is thought to be driven by extracellular stimuli, and among them transforming growth factor-(TGF)β1 often plays a major role [5], [6] [7], [8]. Peritoneal mesothelial cells (MCs) constitutively produce low levels of TGF-β1, as well as other factors, such as bone morphogenic proteins (BMPs), that may counteract the induction of EMT [9], [10]. Besides TGF-β1 and BMPs, other stimuli, such as FGF, CTGF, TNFα, IL-1β, HGF as well as extracellular matrix (ECM) proteins such as fibronectin and collagen may play a role in the induction of peritoneal EMT and fibrosis [1]. Overall, the balance between pro- and anti-EMT extracellular factors may determine the status of the MCs. This concept implicates that, at least during the first stages, the epithelial/mesenchymal status of MCs is reversible, and may actively be modified by extracellular factors. To this purpose, the reversal from a mesenchymal to an epithelial status (MET: Mesenchymal to Epithelial transition) has been already demonstrated in MCs [9], [10], [11], and may depend on the presence of proteins of the TGFβ family such as BMP7, HGF, corticosteroids or vitamin D analogues [9], [10], [12], [13].

A common hallmark in EMT studies is the downregulation of epithelial markers. Among them, E-cadherin downregulation is often followed by replacement with more motile cadherins, such as N-cadherin [14]. Transcription factors, such as those of the Snail, Zeb and HLH families, play a major role in transcriptional downregulation of E-cadherin and other genes involved in EMT [15]. Besides E-cadherin, other epithelial markers, such as cytokeratins, become downregulated during EMT whereas proteins related to the mesenchymal state or fibrosis, such as vimentin, collagens, fibronectin and PAI-1 are upregulated [6], [7].

Many signalling pathways are involved in the induction of EMT and its reversal. Smad2–3 are directly induced by TGF-β1 and have a primary role in peritoneal EMT and fibrosis. Actually, the balance between TGF-β1 activated Smad2–3 and BMP-activated Smad1–5–8 controls the EMT status of the cell in many experimental systems [9], [16]. Besides this, the induction of EMT and fibrosis may be regulated by Smad-independent pathways, such as GSK3β, β-catenin, NF-κB, PI3K, ERK, p38 and JNK MAPK [7], [17], [18], [19].

TGFβ-activated kinase (TAK)1, also known as MEKK7, is a member of the MAPKKK family and is activated by different stimuli, such as BMPs, Toll like receptors (TLR), IL-1β and TNFα [20], [21] TAK1 is involved in the regulation of innate and adaptive immunity, in vascular development and keratinocyte, hematopoietic and hepatocyte survival [22]. TAK1 activation involves the binding to the TAK-associated binding protein 1 (TAB1), TAB2 and TAB3 and interaction with E3 ubiquitin ligase TRAF6, that directly interacts with a consensus motif present in TGFβRI [23]. The TGFβRI-TRAF6 interaction is required for TGFβ-induced autoubiquitylation of TRAF6 and subsequent activation of p38 and JNK, through activation of their intermediate kinases MKK3/MKK6, and of NF-κB pathway [24]. Despite the ability of TAK1 in transducing signals from a wide array of inflammatory and pro-fibrotic stimuli, an extensive study on its role in the establishment and maintenance of EMT and fibrosis has not been performed so far. We have recently demonstrated that TAK1 controls E-cadherin downregulation and the acquisition of a spindle-like cellular shape in MCs stimulated with TGF-β1 in combination with IL-1β [19]. Here, we demonstrate that TAK1 is a key regulator of EMT and fibrosis in MCs, and its inhibition mediates MET. TAK1 controls the down-regulation of E-cadherin and the induction of mesenchymal markers and proteins implicated in fibrosis such as N-cadherin, type I collagen, fibronectin and PAI-1. Moreover, wound healing and invasion in *in vitro* assays were reduced upon TAK1 inhibition. TAK1 controls the activity of different transcription factors such as NF-κB, Snail, AP-1, as well as Smad2–3/Smad1–5–8 balance. Thus, TAK1 is at the cross-road of different receptors involved in inflammation and EMT, and is upstream of relevant pathways involved in EMT regulation. Given that EMT of MCs is central to the onset of peritoneal fibrosis and angiogenesis, and that there is no effective treatment for the progressive loss of peritoneal dialytic capacity in patients undergoing peritoneal dialysis (PD), these results provide possible routes for therapeutic intervention.

## Results

### TAK1 inhibition leads to the reversal of the EMT phenotype (MET) in MCs derived from peritoneal effluent of PD patients

**Figure 1:**
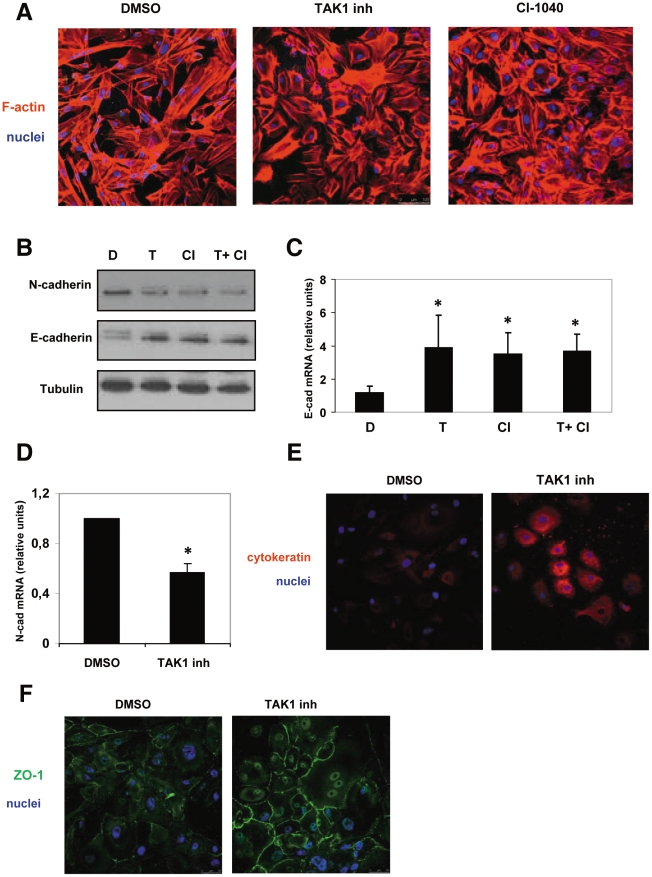
TAK1 inhibition leads to the reversal of the EMT phenotype (MET) in MCs derived from peritoneal effluent of PD patients. **A**, Confocal immunofluorescence analysis (red) of MCs from a patient undergoing PD. Cells were treated with DMSO, NP-009245, (600 nM), CI 1040 (2 µM) for 72 h. Cells were fixed and stained with rhodamine phalloidin. Nuclei were stained with Hoechst 33342 (blue). The immunofluorescence shown is representative of three independent experiments. **B**, Western blots showing the expression of E-cadherin in total cell lysates of MCs. Cells were treated with DMSO, NP-009245 (T) (600 nM), CI 1040 (CI) (2 µM), or a combination of the two pharmacological inhibitors for 72 h. Expression of tubulin was detected as a loading control. **C**, Effect of TAK1 pharmacological inhibition on E-cadherin mRNA expression in MCs from patients undergoing PD. Cells were treated as in A. Quantitative RT-PCR was performed on total RNA. Histone H3 mRNA levels were used for normalization. Bars represent means+s. e. m. of duplicate determinations in three independent experiments. **D**, Effect of TAK1 pharmacological inhibition on N-cadherin mRNA expression in MCs from patients undergoing PD. Cells were treated with DMSO or NP-009245, (600 nM) for 72 h. Quantitative RT-PCR was performed on total RNA. Histone H3 mRNA levels were used for normalization. Bars represent means+s. e. m. of duplicate determinations in three independent experiments. **E** and **F**, Confocal immunofluorescence analysis of MCs from patients undergoing PD. Cells were treated with DMSO or NP-009245, (600 nM) for 72 h. Cells were fixed and stained with a monoclonal antibody against cytokeratin (red) (**D**) or a polyclonal antibody against ZO-1 (green) (**E**). Nuclei were stained with Hoechst 33342 (blue). All data are representative of at least three independent experiments. * p<0.05 versus DMSO treated cells.

To analyze the role of TAK1 in the EMT of MCs, we used MCs isolated from peritoneal effluent of 20 PD patients that have already undergone EMT in vivo (**Table 1**). These cells are often distinguished in epithelioid, and non epithelioid, and their stage of transdifferentiation correlates with the progression of peritoneal damage and the time of PD treatment [25]. We detected basal level of endogenous TAK1 phosphorylation in these cells, which was greatly enhanced by treatment with okadaic acid (OA) (**Figure S1A**). To inhibit TAK1 basal activity, we treated these cells with a specific pharmacological inhibitor (NP-009245, 600 nM) for 72 h [26]. This is a derivative of 5Z-7-oxozeanol, a pharmacological compound that has been recognized as a selective and irreversible inhibitor of TAK1 ATP binding in *in vitro* studies [26]. Interestingly, we observed that treatment with NP-00924 also decreases TAK1 expression (**Figure S1B**). TAK1 inhibition led to the loss of spindle-like shape and to a partial reacquisition of the ‘cobblestone’ epithelial-like phenotype (**Fig. 1A**). The inhibition of ERK1/2 pathway, which causes EMT reversal [18], led to similar results. To inhibit ERK1/2 pathway we used CI-1040, an highly specific allosteric inhibitor of MEK1 activity [27]. TAK1 inhibition increased E-cadherin, as shown in a western blot analysis (**Fig. 1B**) and in RT-PCR (**Fig. 1C**), while reducing N-cadherin mRNA (**Fig. 1D**) and protein (**Fig. 1B**) expression. The simultaneous treatment with MEK1 and TAK1 pharmacologic inhibitors did not further increase E-cadherin levels, with respect to the TAK1 inhibitor alone (**Fig. 1C**). Also, treatment with both pharmacological inhibitors did not increase the reacquisition of a ‘cobblestone’ phenotype (data not shown). TAK1 inhibition led also to an increment of cytokeratin expression (**Fig. 1E**), and to an increased ZO-1 localization at intercellular junctions (**Fig. 1F**).

**Table 1:** 

FIG.		1A	1B	1C	1D	1E	1F	2A	2B	2C	2D	2E	3A	3B	3C	3D	4A	4B	4C	4D	4E	5A	5B	S1	S2
N.	NE	X				X		X																	
1	NE	X				X	X	X																	
2	E	X				X	X																		
3	E		X	X																					
4	E		X	X																					
5	NE		X	X																					
6	E				X				X	X											X				
7	E				X				X	X											X				
8	NE				X				X	X											X				
9	E						X	X																	
10	NE						X	X																	
11	NE										X	X													
12	NE										X	X													
13	E										X	X													
14	E										X	X													
15	E																					X	X		
16	E																					X	X		
17	NE																					X	X		
18	NE																					X	X		
19	NE																					X	X		
20	E												X	X	X	X									
HPMC1													X	X	X	X									
HPMC2													X	X	X	X									
HPMC3																			X						
HPMC4																			X						
HPMC5																			X						
MeT5A																	X	X		X					

### TAK1 inhibition limits the expression of mesenchymal markers by MCs derived from peritoneal effluent of PD patients

**Figure 2:**
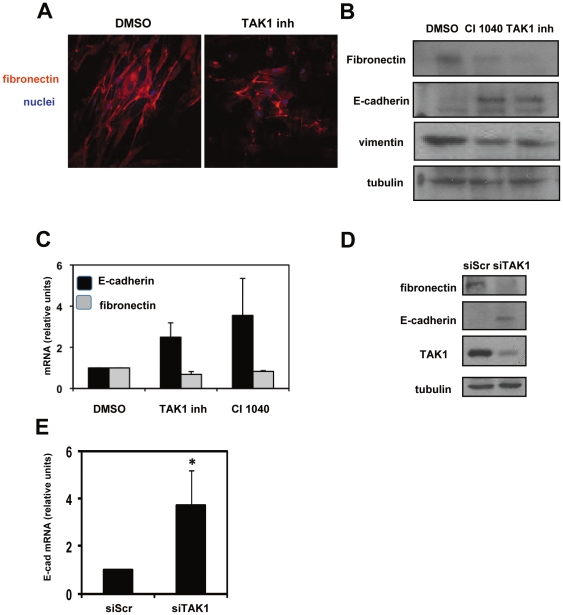
TAK1 inhibition limits the expression of mesenchymal markers by MCs derived from peritoneal effluent of PD patients. **A**, Confocal immunofluorescence (red) of MCs from a patient undergoing PD. Cells were treated with DMSO or NP-009245 (600 nM) for 72 h. Cells were fixed and stained with a monoclonal antibody against fibronectin. Nuclei were stained with Hoechst 33342 (blue). The immunofluorescence shown is representative of three independent experiments. **B**, Western blots showing the expression of fibronectin, E-cadherin and vimentin in total cell lysates of MCs treated with DMSO, NP-009245 (600 nM) or CI-1040 (2 µM) for 72 h. Expression of tubulin was used as a loading control. The results shown are representative of three independent experiments. **C**, Effect of TAK1 pharmacological inhibition on fibronectin and E-cadherin mRNA expression in MCs from patients undergoing PD. Quantitative RT-PCR was performed on total RNA from MCs treated as in A. Histone H3 mRNA levels were used for normalization. Bars represent means+s. e. m. of duplicate determinations in three independent experiments. **D**, Western blots showing the expression of E-cadherin, fibronectin and TAK1 in total cell lysates of MCs transfected with either control or specific TAK1-targeting siRNAs. Scr, cells transfected with control siRNA. Data are representative of three independent experiments. **E**, Effect of TAK1 siRNA silencing on E-cadherin mRNA expression in MCs. Cells were treated as in D. Quantitative RT-PCR was performed; bars represent means+s. e. m. of duplicate determinations in three independent experiments. * p<0.05 versus siScrambled treated cells.

Next, we asked whether TAK1 may also control the expression of mesenchymal markers in MCs from peritoneal effluent. MCs produced bundles of fibronectin, which were reduced upon TAK1 inhibition with pharmacological inhibitor (**Fig. 2A**). WB and RT-PCR analysis (**Fig. 2B–C**) confirmed the reduction of fibronectin at protein and mRNA levels. Vimentin was also reduced in MCs treated with TAK1 inhibitor (**Fig. 2B**). Mesothelial cells, due to their mesenchymal origin, express constitutively vimentin, and its levels increase upon EMT induction [2]. We confirmed the effect of TAK1 on E-cadherin by specific knockdown. After TAK1 knockdown, E-cadherin protein (**Fig. 2D**) and mRNA (**Fig. 2E**) levels were markedly increased. Also, fibronectin expression was reduced upon specific TAK1 silencing (**Fig. 2D**). These results suggest that TAK1 inhibition may counteract the *in vivo* EMT of MCs from peritoneal effluent of patients undergoing PD. TAK1 inhibition promotes the reversal to a ‘cobblestone’ phenotype, the increase of epithelial markers, such as E-cadherin, cytokeratin, the membrane localization of ZO-1, and the decrease of markers associated EMT and fibrosis, such as N-cadherin, vimentin and fibronectin.

### TAK1 inhibition blocks the EMT in MCs induced *in vitro* by a combination of TGF-β1 and IL-1β

**Figure 3:**
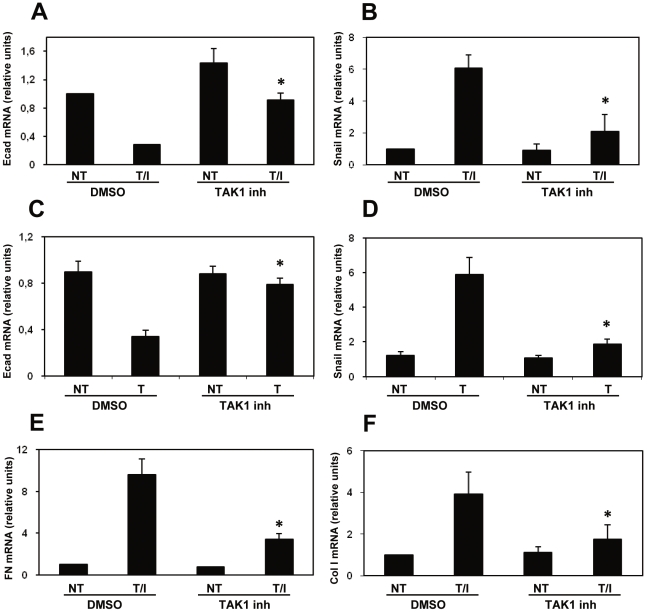
TAK1 inhibition limits the induction of EMT in HPMCs treated with TGF-β1 in combination with IL-1β. Effect of TAK1 pharmacological inhibition on E-cadherin (**A, C**), Snail (**B, D**), fibronectin (**E**) and type I collagen (**F**) mRNA expression in HPMCs. Quantitative RT-PCR was performed on total RNA from MCs pre-treated with DMSO, or NP-009245, (600 nM) for 1 h and then left untreated or stimulated with TGF-β1 0.5 ng/ml) in combination with IL-1β 2 ng/ml) (T/I) or TGF-β1 alone at the same concentration (T) for 24 h. Histone H3 mRNA levels were used for normalization. Bars represent means+s. e. m. of duplicate determinations in three independent experiments. * p<0.05 versus DMSO treated cells.

We then studied the role of TAK1 in the induction of EMT *in vitro* upon stimulation of primary MCs from human omentum (HPMCs) with TGF-β1 in combination with IL-1β for 24 h. Under these conditions, these cells acquire a spindle-like phenotype, undergo E/N-cadherin switch and increase FN and vimentin expression [2], [18], [19]. TAK1 inhibition limited E-cadherin mRNA downregulation upon T/I induction in these cells (**Fig. 3A**). Interestingly, E-cadherin downregulation inversely correlated with Snail expression (**Fig. 3B**). TAK1 inhibitor exerted an analogous effect in the modulation of E-cadherin and Snail levels when EMT was induced using TGF-β1 alone (**Fig. 3C–D**). TAK1 inhibition also reduced fibronectin (**Fig. 3E**) and type I collagen expression in RT-PCR experiments (**Fig. 3F**). These results demonstrate that TAK1 inhibition blocks *in vitro* EMT induction in MCs treated with TGF-β1 alone or in combination with IL-1β.

### TAK1 controls the activation of multiple transcription factors relevant for the induction of EMT and fibrosis

**Figure 4:**
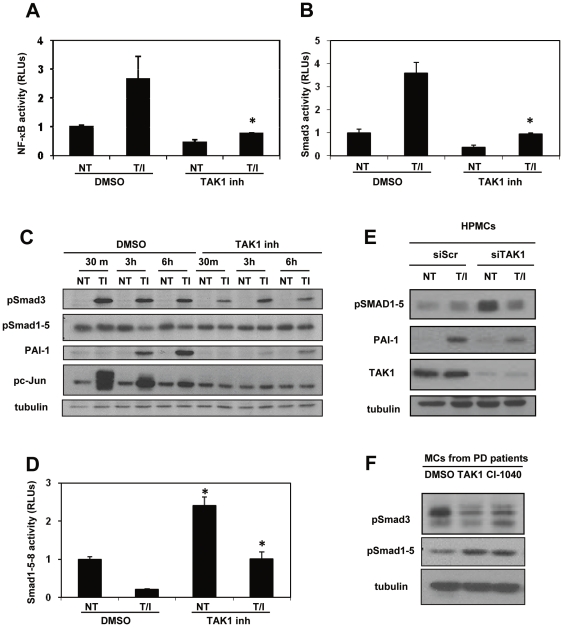
TAK1 controls the activation of multiple transcriptional factors relevant for the induction of EMT and fibrosis. **A**, Effect of TAK1 inhibition on NF-κB transcriptional activity. MeT-5A cells were transiently transfected with the KBF-luc reporter plasmid together with Renilla luciferase. Cells were pretreated with NP-009245 (600 nM) for 12 h and then left untreated (NT, gray bars) or co-stimulated for 9 h with TGF-β1 (0.5 ng/ml) in combination with IL-1β (2 ng/ml) (T/I). Bars represent means+s. e. m. of triplicate determinations in three independent experiments. **B**, Effect of TAK1 inhibition on PAI-1 transcriptional activity. MeT-5A cells were transiently transfected with a PAI-1 reporter plasmid together with a Renilla luciferase-coding plasmid. Cells were stimulated as above. Bars represent means+s. e. m. of triplicate determinations in three independent experiments **C**, Western blots showing the expression of phospho-Smad3, phospho-Smad1–5, PAI-1 and phospho-c-jun in total cell lysates of HPMCs treated for different times with TGF-β1 0.5 ng/ml) in combination with IL-1β (2 ng/ml) (T/I). Expression of tubulin was used as a loading control. The results shown are representative of three independent experiments. **D**, Effect of TAK1 inhibition on Smad1–5–8 transcriptional activity. MeT-5A cells were transiently transfected with the BRE-luc reporter plasmid together with a Renilla luciferase-coding plasmid. Cells were pretreated with NP-009245 (600 nM) for 12 h and then left untreated (NT, gray bars) or co-stimulated for 12 h with TGF-β1 (0.5 ng/ml) in combination with IL-1β (2 ng/ml) (T/I). Bars represent means+s. e. m. of triplicate determinations in three independent experiments. **E**, Western blots showing the expression of phospho-Smad1–5, PAI-1 and TAK1 in whole cell lysates of HPMCs transfected with either control or specific TAK1-targeting siRNAs and then left untreated or stimulated for 4 h with TGF-β1 (0.5 ng/ml) in combination with IL-1β (2 ng/ml) (TI). Scr: cells transfected with control siRNA. Data are representative of three independent experiments **F**, Western blot showing the expression of phospho-Smad1–5–8 in MCs from a patient undergoing PD. Cells were treated with DMSO, CI 1040 (2 µM), NP-009245, (600 nM) for 24 h. Expression of tubulin was used as a loading control. All data are representative of at three independent experiments. * p<0.05 versus corresponding sample treated with DMSO.

We next analysed signalling pathways induced by TAK1 that may be relevant for the induction of EMT and fibrosis in MCs. We have already demonstrated a major role for the ERK1/2-NF-κB pathway in the induction and maintenance of EMT in MCs [18]. As expected, TAK1 inhibition led to a markedly reduced NF-κB transcriptional activity in MeT-5A cells in basal conditions and upon stimulation with TGFβ in combination with IL-1β (T/I) (**Fig. 4A**). We analyzed Smad3 activity, which is involved in the onset of peritoneal fibrosis after exposure to TGF-β1 [28]. TAK1 inhibition led to reduced PAI-1 promoter activity, which is controlled by Smad3 [29] (**Fig. 4B**). Moreover, TAK1 inhibition led to a reduction of both c-jun and Smad3 c-terminal phosphorylation after T/I stimulation in MCs (**Fig. 4C**). In addition, PAI-1 protein levels were markedly reduced upon TAK1 inhibition (**Fig. 4C**). Interestingly, Smad1–5–8 transcriptional activity, as well as Smad1–5 c-terminal phosphorylation, were enhanced by TAK1 inhibition (**Fig. 4C–D**). Also, specific TAK1 silencing led to increased Smad1–5 phosphorylation and reduced levels of PAI-1 expression (**Fig. 4E**). These results were confirmed using MCs from PD patients. Treatment of these cells with TAK1 and MEK1 inhibitors led to enhanced Smad1–5, and reduced Smad3 phosphorylation (**Fig. 4F**). Overall, these results suggest that TAK1, trough the activation of multiple transcription factors, is at the cross-road of signalling pathways controlling EMT and fibrosis.

### TAK1 controls directed motility and invasion of MCs from peritoneal effluent

**Figure 5:**
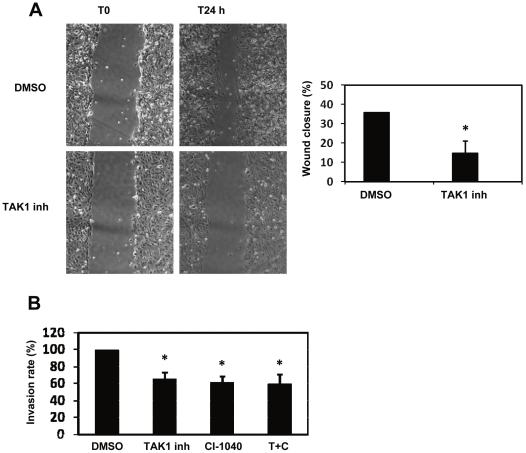
TAK1 controls wound closure and invasion on type I Collagen gel in MCs from peritoneal effluent. **A**, Effect of TAK1 inhibition on wound closure. Left, MCs from a patient undergoing PD were allowed to reach 100% confluency in a 6 well plate. MCs were pre-treated with DMSO or NP-009245 (600 nM) for 24 h in culture medium supplemented with 10% FCS. A scratch wound was created on the cell surface using a micropipette tip. The wound area was photographed every 30 min for 24 h by bright field microscopy (4× magnification). The width of the wound was measured and the wound closure rate was calculated. 9 different points from 3 different scratches were analyzed per condition. Only the beginning and the end of the test analysis is shown. The result described is representative of 5 independent experiments. Right, quantification of the experiment described before. P *<0.05 compared to DMSO-treated cells. **B**, Effect of TAK1 and MEK1 inhibition on MCs invasion on type I Collagen gel. MCs were pre-treated with DMSO, NP-009245 (600 nM) or CI-1040 (2 µM) for 24 h. Cells were allowed to invade type I Collagen gels for 24 hours. Invading cells were fixed and nuclei were counted. Nuclei were counted in ten fields per sample using a fluorescence microscope (40× magnification). Each experiment was carried out in duplicate, and at least 5 experiments were performed. P *<0.05 compared to DMSO-treated cells.

We last asked whether TAK1 inhibition may affect functional events connected with EMT. Increased cell motility and invasion are major features of cells undergoing EMT, even in MCs, that have been demonstrated to invade the submesothelial stroma during transdifferentiation [2], [28], [30]. A transient EMT takes place *in vivo* during wound healing in epithelial cells [31]. We mimicked this physiopathologic condition performing an *in vitro* scratch assay on confluent monolayers of MCs from PD patients. Pre-treatment with TAK1 inhibitor significantly reduced the closure of the scratch (**Fig. 5A**). Moreover, cell invasion through type I collagen gels was reduced upon TAK1, as well as MEK1 inhibition (**Fig. 5B**). Interestingly, the simultaneous treatment with MEK1 and TAK1 pharmacologic inhibitors did not further reduce MCs invasion. These results demonstrate that TAK1 controls MCs directed migration during scratch closure as well as invasion, which are important features of the EMT program.

## Discussion

In this study, we analyzed the role of TAK1 in the induction and maintenance of EMT in MCs from peritoneal effluent of patients undergoing PD. By means of morfological, biochemical and functional analyses, we demonstrated that the inhibition of TAK1 activity induces reversal towards the epithelial phenotype, and we provided mechanistic insight about it.

In the peritoneum exposed to PD fluids, recurrent low grade inflammatory cytokines such as TGF-β1 and IL-1β are actively released, triggering pro-inflammatory and pro-fibrotic signals that may ultimately lead to ultrafiltration failure and fibrosis [9] [2]. These and other cytokines may be produced by mesothelial cells, that, as well as resident peritoneal macrophages, contribute to the maintenance of an extracellular environment favourable to the EMT establishing or maintenance. Interestingly, this ‘pro-EMT’ milieu may be maintained also *in vitro*, since MCs produce soluble factors, like M-CSF, that induce surviving of contaminating macrophages in mesothelial cell cultures [32], that may contribute in sustaining EMT status of MCs. In support of our hypothesis of an auto-perpetuating loop in MCs that have undergone EMT, we found TAK1 basally activated in MCs from PD patients. TAK1 activity is very labile, since it is tightly controlled by phosphatases, and p38 MAPK has a role in this regulation [19].

By using a specific pharmacological inhibitor, as well as gene silencing, we found that TAK1 inhibition led to the reacquisition of an apical-basolateral cell polarity, i.e. the reversal to a ‘cobblestone’ phenotype. Besides these morphological changes (that are a first requirement for the definition of EMT), TAK1 inhibition led to an increased expression of epithelial markers, such as E-cadherin and cytokeratins, increased ZO-1 membrane localization, as well as downregulation of mesenchymal markers, such as vimentin and N-cadherin. TAK1 inhibition led to the same effect both in cells that had already undergone EMT *in vivo* and in cells where EMT was induced by stimulation with TGF-β1 in combination with IL-1β. Moreover, TAK1 inhibition decreased the expression of fibronectin, collagen1 and PAI-1. Fibronectin and collagen1 are main components of the ECM and their expression may induce activatory loops in MCs via interaction with β1 and β1 integrins in MCs [2]. PAI-1 is often linked to the induction of fibrosis, since its expression leads to a decrease of plasmin and other protease activities, thus stabilizing the ECM [33]. Besides this effect, PAI-1 may induce fibrosis favouring cell detachment and interstitial migration of macrophages and myofibroblasts [34]. This may have a role also in the case of MCs, when invading the sub-mesothelial spaces during PD. There are conflicting results about the ability of TAK1 in the induction of fibrosis. Previous *in vitro* studies demonstrated a positive role of TAK1 in inducing fibronectin and collagen expression in different cell lines [35] however, a recent *in vivo* report demonstrated increased liver fibrosis subsequent to spontaneous hepatocyte cell death upon genetic disruption of TAK1 [36]. Our results, obtained in a peculiar *ex vivo* setting, clearly appoint for a positive role of TAK1 in inducing fibrosis in the peritoneal membrane.

Our *in vitro* results, obtained by MCs stimulation with TGF-β1 in combination with IL-1β coincide with previous studies where, in a model of lung fibrosis, adenovirus mediated TGF-β1 gene transfer induced severe and progressive fibrosis without apparent inflammation, whereas IL-1β induced inflammation and progressive fibrosis linked to IL-1β-dependent TGF-β1 expression [37]. To this respect, IL-1β effect was different from TNFα which induced strong inflammation without subsequent fibrosis. MCs from lung and peritoneum show a striking similarity. In our experimental system, combined stimulation with TGFβ1 and IL-1β has additive effects on cell morphology and cell migration [2], E-cadherin downregulation [18] NF-κB nuclear translocation [18].

Moreover, combined stimulation with TGFβ1 and IL-1β leads to increased ERK1/2 phosphorylation (**Figure S2A**) and increased Smad3 transcriptional activity (**Figure S2B**). There are several points of cross-talk between signaling pathways activated by TGF-β1 and IL-1β [38], and their combined use provides a more physiological analysis for specific biological responses. Thus, our experimental system unifies the different proinflammatory and profibrotic insults present in injured peritoneum in a unique pathological model, and emphasize the role of TAK1 as common inducer of peritoneal fibrosis and EMT. However, the fact that TAK1 inhibition is able to block the EMT induced by TGFβ1 alone demonstrates that TAK1 controls a TGFβ1 dependent pathway that is independent on the inflammation.

In our system, TAK1 regulates the expression of epithelial and fibrotic markers essentially at the level of mRNA expression, since we found concordance between mRNA and protein expression of E-cadherin, type I collagen, and fibronectin. We thus analyzed the role of TAK1 in the induction of different transcription factor activities. TAK1 controls the activation NF-κB, c-jun and Smad3. All these transcription factors have been shown to induce EMT and fibrosis [39] [40], [41]. In particular, PAI-1 transcription is mainly induced by Smad3 and AP-1. Interestingly, TAK1 may affect both transcription factors since it controls c-jun phosphorylation, which is a component of AP-1 complex. However, these transcriptional factors may act in synergy, since many studies have reported a cooperation among different transcription factors in the induction of transcriptional activity. Smad3–4 may form a transcriptional repressor complex with Snail that mediate EMT in tumours [42]. Functional and physical interactions between Smad3–Smad4 and c-Jun-c-Fos may mediate TGFβ induced transcriptional activity [43] [44], [45].

It has been widely demonstrated that TAK1 controls NF-κB and c-jun [21], whereas TAK1 activity on the Smads is object of controversy. TAK1 is not required for TGFβ induced Smad2 activation [22]; TAK1 may directly phosphorylate Smad1 at c-terminus, thus enhancing its activity [46]. The reported differences about the effect of TAK1 may refer to different experimental systems used. In our case, TAK1 inhibition led to a decrease of Smad3 c-terminal phosphorylation, which is linked to transcription activity. Moreover, TAK1 may also affect Smad3 activity indirectly, inducing the degradation of the inhibitor SnoN [47], that counteracts Smad3 nuclear activity.

Besides controlling Smad3 activity, TAK1 may have a broader effect on the balance between Smad2–3 and Smad1–5–8, that plays a major role in EMT status [6]. In our system, TAK1 inhibition led to an increased transcriptional activity of Smad1–5–8 as measured as BRE-luc promoter activation, as well as increased c-terminal Smad1–5 phosphorylation.

Our previous study demonstrated that ERK1/2 inhibition was permissive for EMT reversal in cells from PD patients [18]. We analyzed whether in our experimental model ERK1/2 and TAK1 may act independently or have common downstream targets. In some experiments, we treated MCs simultaneously with inhibitors of both MEK1 and TAK1. MEK1 inhibition along with TAK1 inhibition did not have additional effects in E-cadherin or fibronectin expression, nor in morphological changes. This suggests that ERK1/2 and TAK1 activities may converge to the same common targets. We identified in a previous study an ERK1/2/NF-κB/Snail pathway controlling EMT in MCs where both TAK1 and ERK1/2 activities may converge [18], although we do not exclude that these two kinases may have specific effects.

We last focused on the functional role of TAK1 in MCs motility. MCs invasion through the basal lamina to the submesothelial stroma has a major role in the induction of vasculopathy and fibrosis [1] [28]. On the other hand, directed migration of MCs may be useful to repopulate areas of the peritoneal membrane that become devoid of cells, especially after peritonitis episodes [48].

We performed an *in vitro* scratch assay on MCs monolayer and we found that TAK1 inhibition significantly reduced closure of the scratch in a MCs monolayer as well as invasion through collagen gel. These results suggest that TAK1 may be functionally implicated in the increased migration and invasion of MCs during EMT, but further in vivo analyses would be needed to elucidate the role of TAK1 in the migration of MCs.

**Figure 6:**
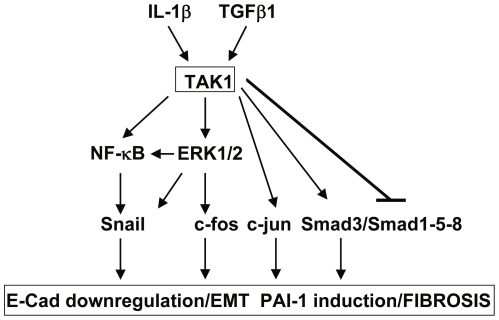
TAK1 as a checkpoint of major signalling pathways controlling EMT and fibrosis. Rapidly induced by a wide array of pro-inflammatory and pro-fibrotic stimuli, TAK1 activation plays a central role in the induction of the EMT program. Besides controlling classical inflammatory pathways, such as NF-κB and MAPKs, TAK1 activation affects Smad3/Smad1–5 balance. TAK1 activation in MCs relies on cadherin switching, PAI-1 expression, increased ECM protein production, as well as the acquisition of invasive abilities. See text for details.

TAK1 was characterized as a kinase induced by TGF family members and also by other inflammatory stimuly such as IL-1β, TNFα and TLR ligands; however, an extensive study on the role of this kinase in the establishment and maintenance of EMT and fibrosis has never been performed. Here, the role of TAK1 in EMT and fibrosis is extensively characterized in a peculiar experimental system based on primary MCs from PD patients that have undergone EMT *in vivo*. We propose TAK1, with its ability in orchestrating the activation of different transcription factors, as a central point of control of EMT and fibrosis (**Fig. 6**). To this respect, this study is relevant both for peritoneal dialysis research and for the basic understanding of molecular mechanisms underlying EMT and fibrosis.

## Materials and Methods

### Isolation and culture of MCs

Effluent-derived MCs were isolated from clinically stable PD patients using a method described previously [49]. Briefly, nocturnal peritoneal dialysates from clinically stable patients are drained and processed immediately. Bags are hanged for 3–4 hours in an incubator at 4°C to facilitate the accumulation of floating cells at the bottom of the bags. The supernatant is then carefully removed by vacuum with a sterile pipette, leaving approximately 200 mL of sediment. The cells are transferred to four 50-mL tubes, centrifuged at 1200 rpm for 10 minutes, and washed twice with phosphate-buffered saline (PBS). The cell pellets are suspended in 5–7 mL of culture medium, counted in a Neubauer chamber, seeded in 25-cm2 tissue culture flasks, and incubated at 37°C in a humidified atmosphere with 5% CO2. Cells were cultured in Earle's 199 medium supplemented with 20% fetal calf serum, 50 U/ml penicillin, 50 µg/ml streptomycin, and 1% Biogro-2 (containing insulin, transferrin, ethanolamine, and putrescine) (Biological Industries, Beit Haemek, Israel), and after 10–15 days cultures reached confluence. The morphological features of cells in confluent cultures remained stable during the two to three passages used for experiments. Confluent MC cultures from PD effluents show one of two major phenotypes, epithelial-like (epithelioid) or non-epithelioid, which remain stable for two to three cell passages [25]. Epithelioid cells have a cobblestone appearance, and they are often morphologically indistinguishable from primary omentum derived mesothelial cells. However, these effluent-derived mesothelial cells with epithelioid morphology already show down-regulation of the epithelial markers E-cadherin and cytokeratins, up-regulation of the mesenchymal marker Snail, and are half as high as HPMC in vertical confocal images, indicating that the E cells have initiated the EMT process (early EMT). On the other hand, effluent-derived non-epithelioid cells have a spindle-like fibroblastic shape, and are in advanced steps of the EMT process (late EMT). These non-epithelioid cells express high levels of mesenchymal molecules including fibronectin and type I collagen, show increased migratory/invasive capacity, and express high amounts of pro-fibrotic and angiogenic factors such as VEGF, TGF-β1, CXCL8. [2] [25] [50].

To control for fibroblast contamination, the purity of HPMC and effluent-derived MC cultures was determined from the expression of the standard mesothelial markers, intercellular adhesion molecule (ICAM)-1 and cytokeratins [1]. MCs expressed high levels of ICAM-1; this allows these cells to be easily distinguished from peritoneal fibroblasts [18]. Moreover, HPMCs express high levels of epithelial cytokeratins, which are slowly downregulated during the EMT process. As shown in **Figure S3**, MCs from PD effluents are still generally expressing cytokeratins, although at low levels. MC cultures are generally negative for the endothelial marker CD31 and the pan-leukocyte marker CD45. When isolating both omentum- and peritoneal effluent-derived MCs, we generally obtain highly purified cell populations, with <5% contaminant cells, as determined by FACS analysis (**Figure S4**). Purified samples with >5% contaminant cells are routinely discarded.

HPMCs were obtained by digestion of omentum samples from patients who were undergoing unrelated abdominal surgery [51]. The samples were digested with a 0.125% trypsin solution containing 0.01% EDTA..Cells were cultured as above. To induce EMT, HPMCs were treated with a combination of human-recombinant TGF-β1 (0.5 ng/mL) and IL-1β (2 ng/mL) (R&D Systems, Minneapolis, MN) as described previously [2] [25]. Although both TGFβ1 and IL-1β separately are able to induce EMT phenotypic changes, combined stimulation induces a more complete EMT [2], [18], [47]. The cytokine doses used are in the range of those detected in peritoneal-dialysis fluids in the presence of peritonitis (Lai et al., 2000) and are similar to those used in previous studies [2], [52]. The study was approved by the ethics committee of the Hospital Universitario de la Princesa (Madrid, Spain). Written informed consent was obtained from both PD patients included in this study, for the use of effluent samples, and from omentum donors prior to elective surgery.

The human MC line MeT-5A (ATCC, Rockville, MD) was cultured in Earle's 199 medium, as above, and stimulated with the same doses of TGFβ1 and IL-1β. MeT-5A is an untransformed MC line, which is often used in peritoneal MC research.

### Antibodies and chemicals

The monoclonal antibody against E-cadherin was purchased from BD (Becton-Dickinson Laboratories, Mountain View, CA); monoclonal antibodies against tubulin, pan-cytokeratin, vimentin, and fibronectin were from Sigma (Saint Louis, MO); Polyclonal antibodies against phospho-Smad3, phospho-Smad1–5, phospho-c-jun, phospho-TAK1 (Thr187), TAK1 were from Cell Signaling (Cell Signaling Technology, Danvers, MA); polyclonal antibody against Smad3 was from Santa Cruz Biotechnology (Santa Cruz, CA). Monoclonal antibody against N-cadherin and polyclonal antibody against ZO-1 were from Zymed (Invitrogen, Carlsbad, CA); monoclonal antibody against CD45-FITC was from BD Bioscience, (San Josè, CA); monoclonal antibody against ICAM-1(HU5/3) was from Dr. Yañez-Mo (Madrid, Spain); Fluor 647–phalloidin and Hoechst 33342 were purchased from Invitrogen (Carlsbad, CA). 9-Epimer-11,12-dihydro-(5Z)-7-oxozeanol was from AnalytiCon Discovery (NP-00924), Potsdam, Germany.Alexa. CI 1040 was from Selleck, Houston, USA.

### Western blotting

Monolayers of MCs were lysed in modified RIPA buffer containing: 50 mM Tris-HCl, pH 7.4; 1% NP-40; 0.1% SDS; 0.25% Nadeoxycholate; 150 mM NaCl; 1 mM EDTA; 1 mM EGTA; 1 mM PMSF; 1 µg/ml each of aprotinin, leupeptin and pepstatin; and 25 mM NaF (all from Sigma). Equal amounts of protein were resolved by SDS-PAGE. Proteins were transferred to Nitrocellulose membranes (Amersham Life Sciences, Little Chalfont, UK) and probed with antibodies using standard procedures. Nitrocellulose-bound antibodies were detected by chemiluminescence with ECL (Amersham Life Sciences).

### Confocal microscopy and immunofluorescence

Cells were fixed for 20 minutes in 3% formaldehyde in PBS, permeabilized in 0.2% Triton X-100/PBS for 5 minutes, and blocked with 2% BSA for 20 minutes. For E-cadherin staining, cells were fixed and permeabilized in ice-cold methanol for 5 minutes. Secondary antibodies (conjugated to Alexa-647, -488 and -541) and Hoechst 33342 were from Pierce Chemical Company (Rockford, IL). Confocal images were acquired using a Leica SP5 spectral confocal microscope. The spectral technology allows discrimination between yellow and green fluorescence.

### Flow Cytometry Analysis

Confluent cultures of effluent-derived MCs were trypsinized, washed and resuspended in PBS. A total of 1×10^5^ cells were incubated with 100 µl of monoclonal antibody against ICAM-1(HU5/3), washed with PBS and then incubated with 100 µl of a 1∶50 dilution of an FITC-conjugated anti–mouse Ig. Alternatively, 1×10^5^ cells were incubated with 100 µl of a 1∶50 dilution of a monoclonal antibody against CD45-FITC for 20 min at 4°C and washed with PBS. Finally, fluorescence was measured using a FACScan® flow cytometer (Becton Dickinson Labware, Lincoln Park, NJ).

### siRNA-mediated TAK1 knockdown

1.2×10^5^ cells were seeded on 24-well plates 24 h prior transfection. Cells were transfected overnight with either 80 pmol ON-TARGETplus SMARTpool against human TAK1 (RefSeq NM_145331) or the same amount of ON-TARGETplus Non-targeting Pool and 1 µl Dharmafect 1 (Dharmacon, Lafayette, CO) in 400 µl antibiotic-free medium per well. Transfections were performed twice with a 48 h-interval. 72 h after the last transfection, knockdown efficiency was determined by western blot and cells were processed as indicated.

### Cell transfection and luciferase assays

NF-κB transcriptional activity was measured by transient transfection of MeT-5A cells with the KBF-luc reporter plasmid and subsequent luciferase activity assay [53]. Smad3 transcriptional activity was measured by transient transfection of MeT-5A cells with the PAI-1 reported plasmid [54] and SMAD1–5–8 with BRE-luc reporter plasmid [55] Briefly, 2×10^5^ cells were transfected with 2 µg of reporter plasmid together with 500 ng of the reporter plasmid pRL-null, which bears a promoter-less Renilla luciferase gene (Promega, Madison, WI). Transfections were performed by incubating cells for 4 hours with a mixture of DNA and lipofectamine at a ratio of 1∶2.5 (Lipofectamine 2000; Invitrogen, Carlsbad, CA, USA) in serum-free medium. After transfection, cells were pretreated overnight with vehicle (DMSO) or U0126 (20 µM). Cells were then stimulated with TGF-β1 and IL-1β for the times indicated. Luciferase activity was measured with the dual-luciferase reporter assay system (Promega) according to the manufacturer's instructions and determined in a Sirius single tube luminometer (Berthold Detection Systems GmbH, Pforzheim, Germany). All experiments were carried out in duplicate.

### Wound healing

MCs from effluent of patients undergoing PD were allowed to reach 100% confluency. MCs were pretreated with DMSO or TAK1 inhibitor (NP-009245, 600 nM) for 24 h in culture medium supplemented with 10% FCS. A scratch wound was created on the cel surface using a micropipette tip. The wound area was photographed by bright-field microscopy every 30 min for 24 h. The width of the wound was measured and the wound closure rate was calculated.

### Invasion assay

Invasion assay was performed using polycarbonate inserts with 8 µm pore size (Costar, Cambridge, MA). The inserts were precoated with 40 µl of solution type I collagen (300 µg/ml) (PureCol, Inamed, Fremont, Canada) and incubated overnight at 37°C to allow gel formation. MCs were pre-treated 24 hours with DMSO, NP-009245 (600 nM) or CI-1040 (2 µM) in 10% FBS M-199 medium. 5×10^4^ MCs in 100 µl assay medium (M-199 0% FBS) were added to the upper chamber. Invasion stimulus (10% FBS) was added to the lower chamber in 600 µl assay medium. MCs were allowed to invade for 24 hours. The inserts were then fixed with 4% paraformaldehyde and non-invading cells on the upper face of the membrane were then removed with a cotton swab, filters were cut and nuclei of invading cells were stained with Hoechst 33342. Invading cells were counted in ten fields per sample using a fluorescence microscope (40× magnifications). Each experiment was carried out in duplicate, and at least 5 experiments were performed.

### Quantitative RT-PCR

Total RNA was extracted with the RNeasy kit (Qiagen GmbH, Hilden, Germany), and the cDNA was obtained from 500 ng of total RNA by using an Omniscript RT kit (Qiagen). Quantitative PCR was carried out in a LightCycler (Roche Diagnostics GmbH, Mannheim, Germany) using a SYBR Green kit (Roche Diagnostics GmbH) and the following specific primer sets: 5′TGAAGGTGACAGAGCCTCTG3′ and 5′TGGGTGAATTCGGGCTTGTT3′ for E-cadherin; 5′GCAAATACTGCAACAAGG3′ and 5′GCACTGGTACTTCTTGACA3′ for Snail1; 5′GCTATGATGAGAAATCAACCG3′ and 5′GCTTCCCCATCATCTCCATTC3′ for Collagen1; 5′ CCTGAAGCTGAAGAGACTTGC3′ and 5′CGTTTCTCCGACCACATAGGA3′ for fibronectin; 5′GAAAACCCTTATTTTGCCCC3′ and 5′CACAGGATCTATTTTTAGCC3′ for N-cadherin;5′AAAGCCGCTCGCAAGAGTGCG3′ and 5′-ACTTGCCTCCTGCAAAGCAC3′ for histone H3 (used for normalization). All experiments were carried out in duplicate. After amplification, the PCR products were confirmed by melting-curve analysis and gel electrophoresis.

### Statistical analysis

Statistical significance was determined with a *t*-test and Mann-Whitney test with Graph pad software. *P* values of <0.05 were considered significant.

## Supporting Information

Figure S1 **TAK1 is endogenously phosphorylated in MCs from peritoneal effluent of patients undergoing PD and its inhibition leads to reduced TAK1 expression.** (**A**) Western blots showing the expression of phospho-TAK1 (Thr187) from total cell lysates of MCs from peritoneal effluent of patient undergoing PD or MeT5A cells. Cells were left untreated or stimulated for 15 minutes with okadaic acid (OA), (100 µM). E: epithelioid; NE: non-epithelioid. Expression of tubulin was used as a loading control. (**B**) Western blots showing the expression of phospho-TAK1 (Thr187) and TAK1 from total cell lysates of MCs from peritoneal effluent of patient undergoing PD. Cells were left untreated or treated for 24 h with NP-009245, (600 µM). E: epithelioid; NE: non-epithelioid. Expression of tubulin was used as a loading control. Two samples from an out of five analyzed are shown.
(TIF)

Figure S2 **ERK1/2 phosphorylation and SMAD3 transcriptional activity is increased upon stimulation with TGF-β1 in combination with IL-1β in MCs.** (**A**) Western blots showing the expression of phosphoERK1/2 in total cell lysates of HPMCs left untreated or stimulated for 24 hours with TGF-β1 (0.5 ng/ml), IL-1β (2 ng/ml), or a combination of both cytokines. Expression of tubulin was detected as a loading control. Data are representative of three independent experiments. (**B**) MeT-5A cells were transiently transfected with a PAI-1 reporter plasmid together with a Renilla luciferase-coding plasmid. Cells were stimulated as above for 9 h. Bars represent means+s. e. m. of a representative experiment of three performed.
(TIF)

Figure S3 **Cytokeratin expression in MCs from peritoneal effluent of patients undergoing PD.** Confocal immunofluorescence analysis of MCs from human omentum or from peritoneal effluent patients undergoing PD. Cells were fixed and stained with a monoclonal antibody against cytokeratin (red). Nuclei are in blue.
(TIF)

Figure S4 **ICAM1 and CD45 expression in MCs from peritoneal effluent of patients undergoing PD.** FACS analysis of MCs from peritoneal effluent of 5 patients undergoing PD. Cells were tripsinized, fixed, and were stained with monoclonal antibodies anti-ICAM1 and anti-CD45.
(TIF)
