# Supplementary figures and images for: Inhibition of Transforming Growth Factor-Activated Kinase 1 (TAK1) Blocks and Reverses Epithelial to Mesenchymal Transition of Mesothelial Cells
Source: PLoS One. 2012 Feb 27;7(2):e31492. doi: 10.1371/journal.pone.0031492 (PMC3288041; doi:10.1371/journal.pone.0031492)

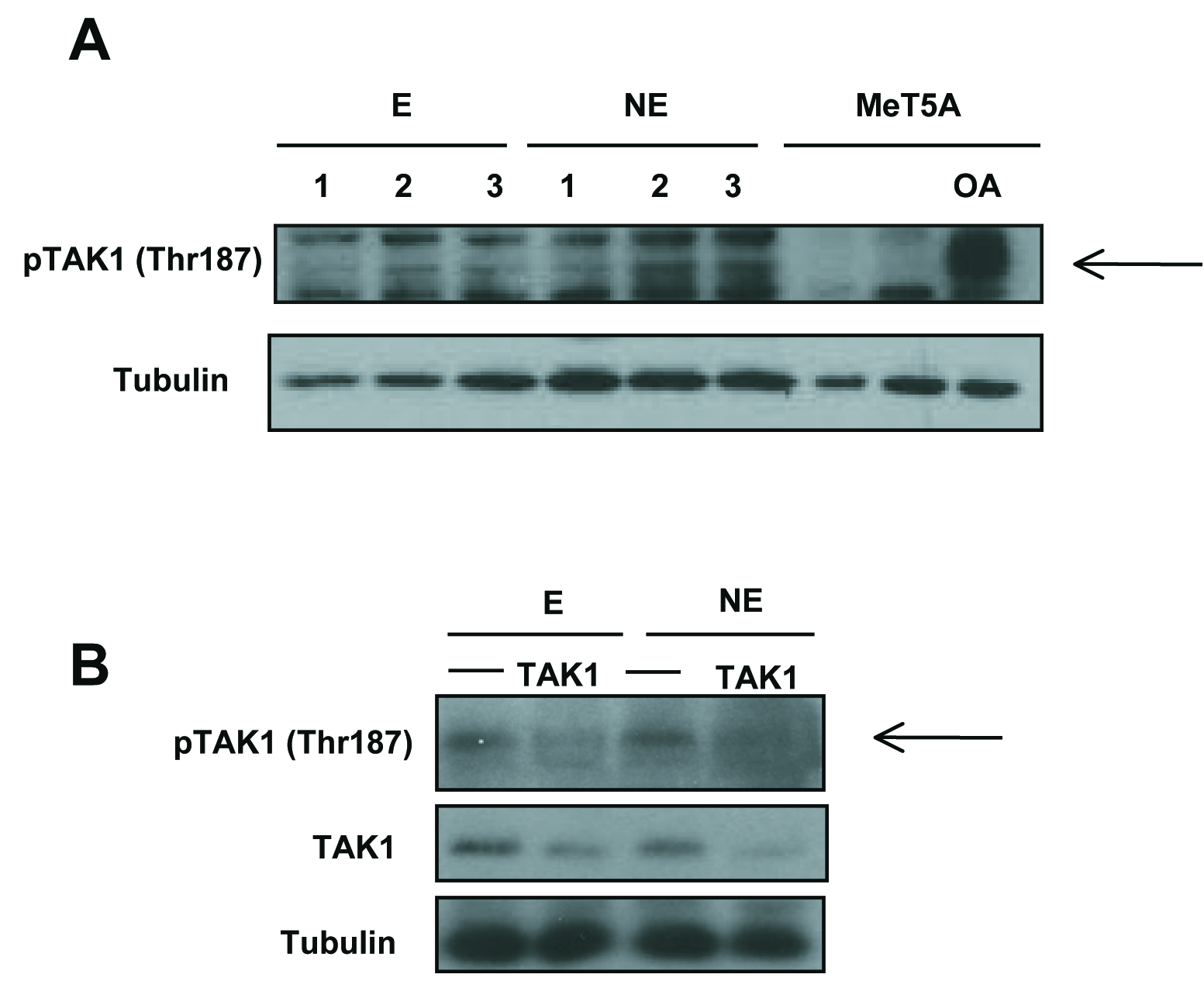

Supplement: Figure S1 — TAK1 is endogenously phosphorylated in MCs from peritoneal effluent of patients undergoing PD and its inhibition leads to reduced TAK1 expression. (A) Western blots showing the expression of phospho-TAK1 (Thr187) from total cell lysates of MCs from peritoneal effluent of patient undergoing PD or MeT5A cells. Cells were left untreated or stimulated for 15 minutes with okadaic acid (OA), (100 µM). E: epithelioid; NE: non-epithelioid. Expression of tubulin was used as a loading control. (B) Western blots showing the expression of phospho-TAK1 (Thr187) and TAK1 from total cell lysates of MCs from peritoneal effluent of patient undergoing PD. Cells were left untreated or treated for 24 h with NP-009245, (600 µM). E: epithelioid; NE: non-epithelioid. Expression of tubulin was used as a loading control. Two samples from an out of five analyzed are shown. (TIF) [file pone.0031492.s001.tif]

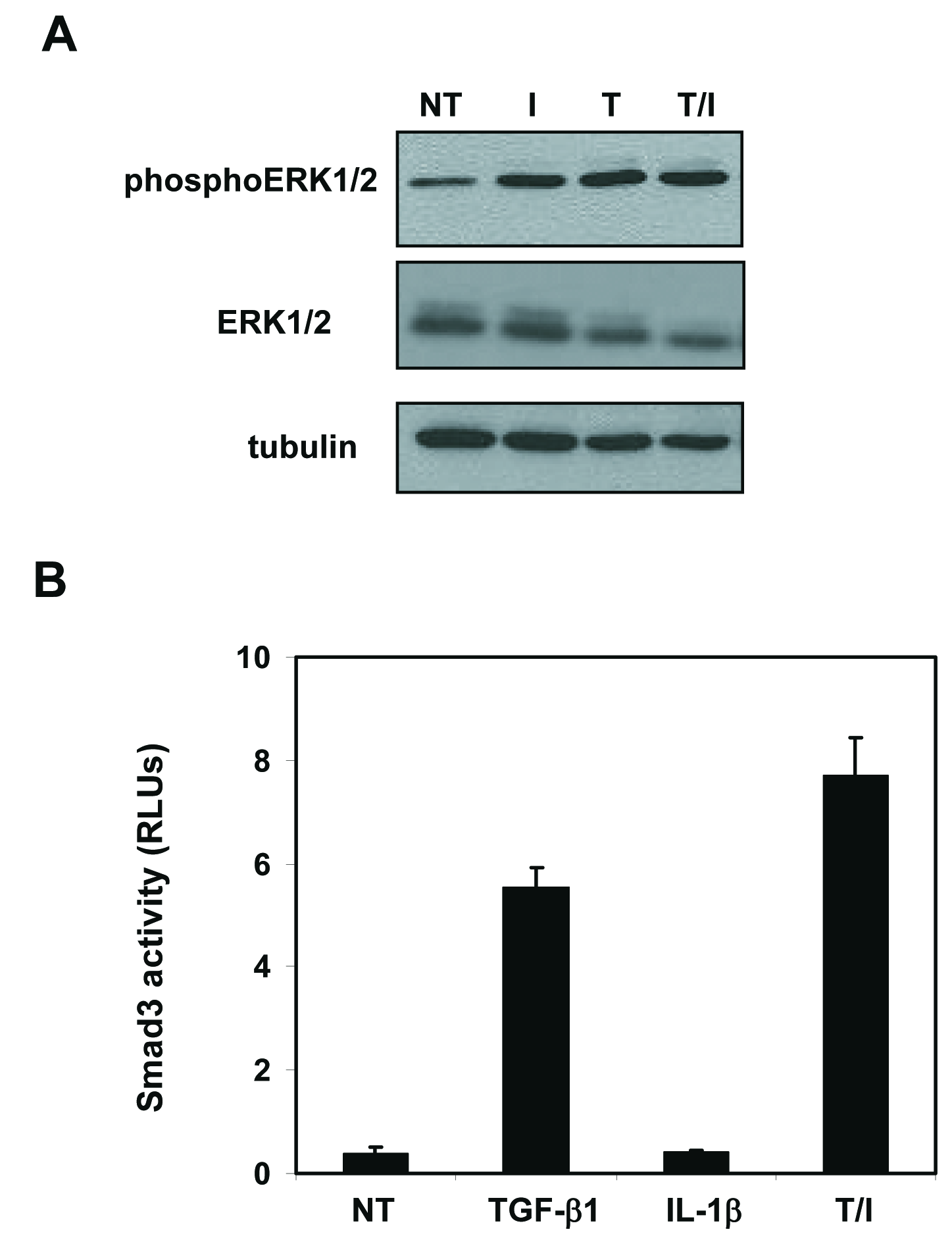

Supplement: Figure S2 — ERK1/2 phosphorylation and SMAD3 transcriptional activity is increased upon stimulation with TGF-β1 in combination with IL-1β in MCs. (A) Western blots showing the expression of phosphoERK1/2 in total cell lysates of HPMCs left untreated or stimulated for 24 hours with TGF-β1 (0.5 ng/ml), IL-1β (2 ng/ml), or a combination of both cytokines. Expression of tubulin was detected as a loading control. Data are representative of three independent experiments. (B) MeT-5A cells were transiently transfected with a PAI-1 reporter plasmid together with a Renilla luciferase-coding plasmid. Cells were stimulated as above for 9 h. Bars represent means+s. e. m. of a representative experiment of three performed. (TIF) [file pone.0031492.s002.tif]

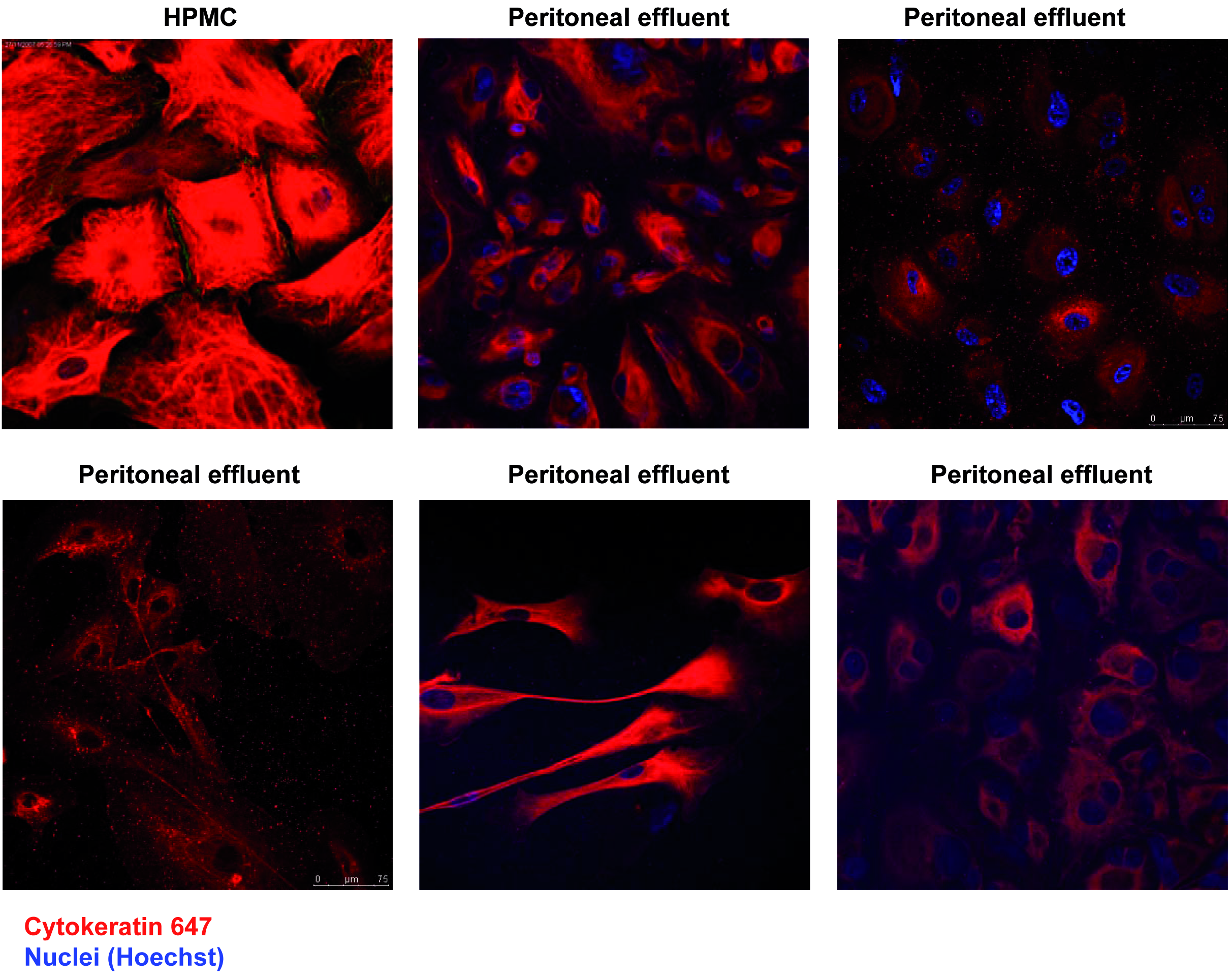

Supplement: Figure S3 — Cytokeratin expression in MCs from peritoneal effluent of patients undergoing PD. Confocal immunofluorescence analysis of MCs from human omentum or from peritoneal effluent patients undergoing PD. Cells were fixed and stained with a monoclonal antibody against cytokeratin (red). Nuclei are in blue. (TIF) [file pone.0031492.s003.tif]

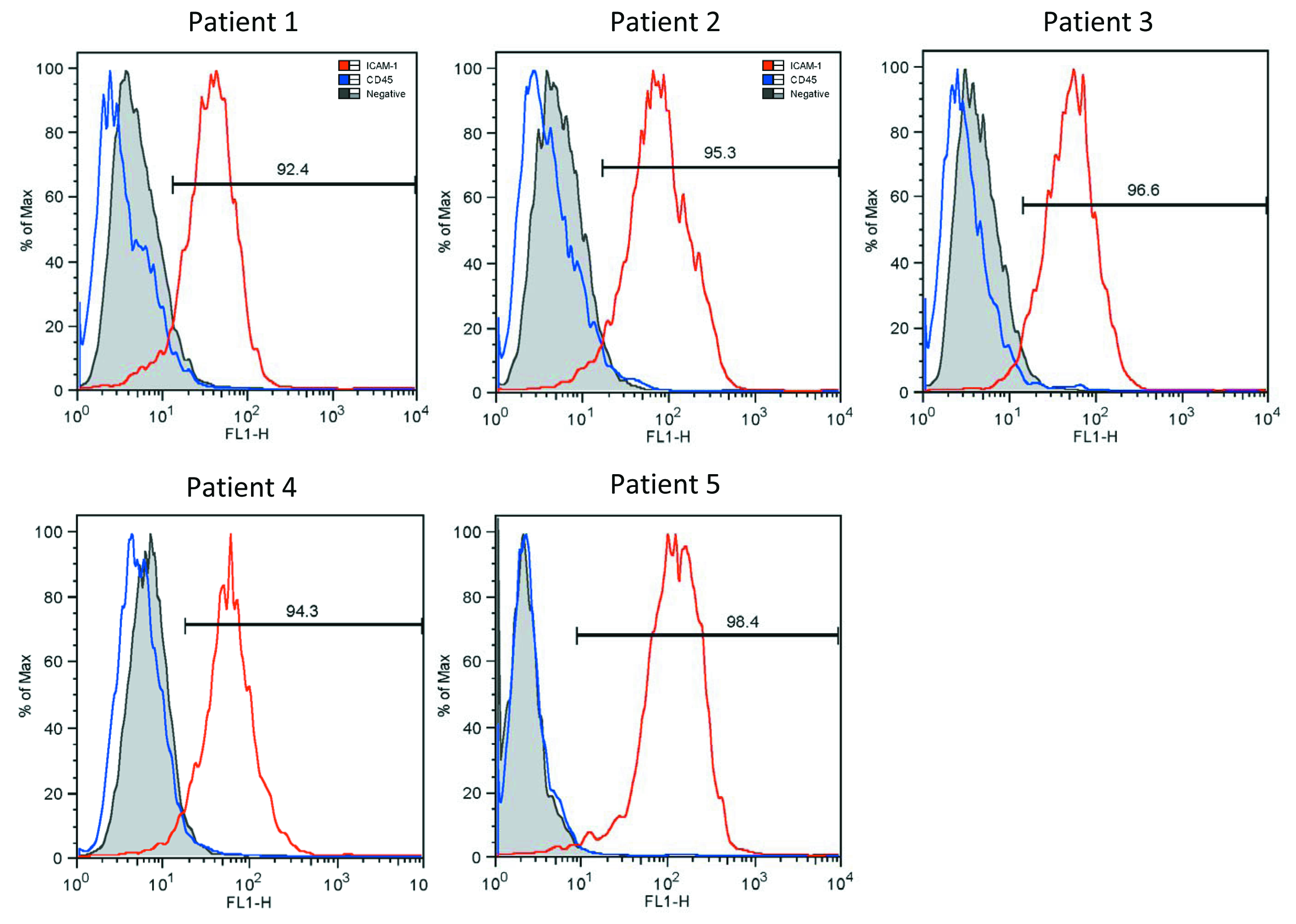

Supplement: Figure S4 — ICAM1 and CD45 expression in MCs from peritoneal effluent of patients undergoing PD. FACS analysis of MCs from peritoneal effluent of 5 patients undergoing PD. Cells were tripsinized, fixed, and were stained with monoclonal antibodies anti-ICAM1 and anti-CD45. (TIF) [file pone.0031492.s004.tif]
